# Efficacy of ^18^F-fluorodeoxyglucose-positron emission tomography/computed tomography in restaging muscle-invasive bladder cancer following radical cystectomy

**DOI:** 10.3892/etm.2015.2187

**Published:** 2015-01-16

**Authors:** HAKAN ÖZTÜRK, İNANÇ KARAPOLAT

**Affiliations:** 1 Department of Urology, School of Medicine, Sifa University, Izmir 35240, Turkey; 2 Department of Nuclear Medicine, School of Medicine, Sifa University, Izmir 35240, Turkey

**Keywords:** bladder cancer, muscle-invasive bladder cancer, cystectomy, positron emission tomography, positron emission tomography/computed tomography, restaging

## Abstract

The aim of the present study was to retrospectively evaluate the contribution and effectiveness of ^18^F-fluorodeoxyglucose-positron emission tomography/computed tomography (^18^F-FDG-PET/CT) scans in the restaging of patients following radical cystectomy due to muscle-invasive bladder carcinoma (MIBC). A total of 51 patients (45 males and six females) who underwent radical cystectomy due to invasive bladder cancer, and had an ^18^F-FDG-PET/CT scan for restaging between July 2007 and April 2013, were included in the present study. The mean age was 62.3±9.79 years (range, 40–82 years). Patients underwent a six-hour fast prior to scanning, and whole-body PET scanning from the skull base to the upper thighs was performed ~1 h after the intravenous injection of 555 MBq ^18^F-FDG. Whole-body CT scanning was performed in a cranio-caudal direction. ^18^F-FDG-PET images were reconstructed using CT data for attenuation correction. Histopathology or clinical follow-up was used to confirm any suspicious recurrent or metastatic lesions. The results for sensitivity, specificity, positive predictive value (PPV), negative predictive value and accuracy of ^18^F-FDG-PET/CT were 92, 83, 94, 77 and 90%, respectively. In conclusion, ^18^F-FDG-PET/CT efficiently detects local recurrence and distant metastases with high sensitivity and PPV in the restaging of patients who underwent radical cystectomy due to invasive bladder cancer. This procedure could play an important role in rendering decisions regarding radiotherapy or chemotherapy and post-operative follow-up, and could influence the entire decision-making process.

## Introduction

Bladder cancer ranks ninth in cancer incidence throughout the world with 380,000 new cases occurring every year. The male to female ratio is 3.8:1 (1). According to the database of Surveillance, Epidemiology and End Results, there has been no significant change in mortality in the last 30 years (2). Bladder cancer is a heterogeneous disease; 70% of patients present with superficial tumours, which tend to recur but are generally not life threatening, and 30% present with muscle-invasive disease, which is associated with a high risk of mortality from distant metastases (3). The transitional cell cancers account for >90% of bladder cancer cases, followed by squamous cell cancer (5%), adenocancers (2%) and undifferentiated cancers occurring in <1% of cases (3). Of the patients that underwent radical cystectomy, 57% had muscle-invasive bladder carcinoma (MIBC) at the time of diagnosis, and the remaining 43% of the cases became muscle-invasive with the progression of the superficial cancer (4). Approximately one-third of patients diagnosed with MIBC have undetected metastases while undergoing treatment for the primary tumour, and a quarter of patients who undergo radical cystectomy also show lymph-node involvement at the time of surgery (5). The standard treatment strategy for MIBC is radical cystoprostatectomy for males and anterior exenteration, including the bladder, urethra, uterus and ventral vaginal wall, for females (3).

For patients with MIBC and nodal dissemination, the frequency of metastasis was shown to be 92% in the regional (perivesical or pelvic), 72% in the retroperitoneal and 35% in the abdominal lymph nodes. A significant correlation was also found between nodal metastases and concomitant distant metastases(6). Standard lymphadenectomy in patients with bladder cancer involves the removal of nodal tissue cranially up to the common iliac bifurcation, with the ureter forming the medial border, and includes the internal iliac, presacral, obturator fossa and external iliac nodes (6). Pelvic lymphadenectomy is a part of the radical cystectomy procedure; however, the borders of lymphadenectomy have not been clearly described in the literature. A number of studies have recommended limited lymphadenectomy but there are also studies suggesting the survival benefit of more extensive lymphadenectomy (5,6). Although radical cystectomy is the preferred treatment for MIBC, metastases develop in ~25% of cases of tumours solely invading the muscular layer and in ~50% of tumours extending into the perivesical tissue (5). Neoadjuvant or adjuvant chemotherapy can be used in the treatment of high-risk patients with invasive bladder cancer; however, systemic chemotherapy is the treatment of choice in metastatic disease.

In the staging of MIBC, magnetic resonance imaging (MRI) is superior to the computed tomography (CT) scan due to a higher resolution in the soft tissue; however, the procedure is accompanied by the disadvantage of low spatial resolution and the side effect of systemic fibrosis. The accuracy of MRI for primary tumour staging varies between 73 and 96% (mean, 85%), which is 10–33% higher (mean, 19%) than that obtained with CT (7). By contrast, CT offers higher sensitivity in extravesical involvement (stages T3a and T3b). The accuracy of CT in determining extravesical tumour extension varies between 55 and 92% and increases with more advanced disease states (8). Pelvic nodes >8 mm and abdominal nodes >10 mm in maximum short-axis diameter, detected by CT or MRI, should be considered to be pathologically enlarged. The sensitivity for the detection of lymph-node metastases is low (48–87%) due to the fact that pelvic lymph-node metastasis is determined based on the size of the lymph nodes on CT and MR images (9). It is well known that metastasis can also occur in normal-sized lymph nodes. Positron emission tomography (PET)/CT scans that combine anatomic and functional images provide more sensitive data in the detection of these lymph nodes.

At present, the detection of distant metastases and local recurrence continues to be a significant problem following radical cystectomy. Furthermore, there is a significant demand for a diagnostic test offering high sensitivity and specificity in predicting residual disease and monitoring the response to treatment following radiotherapy and chemotherapy. ^18^F-fluorodeoxyglucose (^18^F-FDG)-PET/CT is the most important diagnostic tool that allows the processing of functional and anatomical images. The aim of the present study was to retrospectively evaluate the effectiveness and diagnostic role of ^18^F-FDG-PET/CT scans in restaging patients with MIBC who underwent radical cystectomy. The histological findings (where available) or the entire clinical and radiological workup (multidetector computed tomography urography and MRI) were used as a standard reference.

## Materials and methods

### Ethical approval and informed consent

All procedures were performed in accordance with the ethical standards of the World Medical Association committee on human experimentation and with the 1975 Declaration of Helsinki, as revised in 2000. Informed consent was obtained from all patients in the study.

### Patients

A total of 7,938 patients were evaluated and 10,553 ^18^F-FDG-PET/CT scans were performed in the Department of Nuclear Medicine of Sifa University (Izmir, Turkey) between July 2007 and April 2013. In this patient group, 51 patients underwent radical cystectomy with the diagnosis of MIBC and ^18^F-FDG-PET/CT scans were obtained for restaging purposes. The patient population comprised 45 males (88.2%) and six females (11.8%) with a mean age of 62.3±9.79 years (range, 40–82 years).

Thirty patients (58.8%) underwent Bricker ileal conduit urinary diversion, 18 patients (35.2%) underwent the W-configured orthotopic Hautmann ileal neobladder procedure and three patients (6%) underwent ureterocutaneostomy. The results of the pathological and immunohistochemical examinations and data on the histological subtypes of MIBC were available for 48 patients (94%): 47 patients (92.2%) had high-grade transitional cell carcinomas; one (1.9%) had squamous cell carcinoma; and the data on histological subtype for the remaining three patients (5.9%) were not available. The patients were re-assessed with ^18^F-FDG-PET/CT scans for restaging due to suspicion of disease recurrence or for routine follow-up. These patients were retrospectively evaluated, and the pathological findings and ^18^F-FDG-PET/CT data were recorded. Baseline characteristics of the patients are summarised in Table I.

### Imaging and interpretation of data

^18^F-FDG was synthesised using an in-house cyclotron (RDS 111 Cyclotron; Siemens Healthcare, Erlangen, Germany) and an automated synthesis system (CPCU-Chemical Process Control Unit) according to an authorised procedure. ^18^O-H_2_O, used as a target for the synthesis of 18F was supplied by Sharon Marshall Isotops, Ltd. (Tel Aviv, Israel). The patients fasted for five hours, and then their blood glucose level was measured. Each patient was subsequently intravenously injected with 370 MBq ^18^F-FDG. One hour after ^18^F-FDG injection, a CT scan without contrast agent was performed, covering the area from the vertex to the proximal thigh, and the images were used for attenuation correction and image fusion. This was followed by whole-body three-dimensional-PET acquisition with eight bed positions and 3 min emission scan time per position using a dedicated PET/CT scanner (HI-REZ Biograph™ 6; Siemens Healthcare), which provides an in-plane spatial resolution of 4.8 mm and an axial field view of 16.2 cm. The PET data were reconstructed using a Gaussian filter with an ordered-subset expectation maximisation algorithm (three iterations, eight subsets), re-oriented in transverse, coronal and sagittal planes, and assessed through comparisons with corresponding CT images.

A forced diuresis was performed on 18 patients (35.2%) who underwent the orthotopic continent ileal neobladder procedure. Those patients were instructed to drink an additional 500 ml water and to void frequently. Delayed pelvic images were acquired 2.5–3.0 h after injection of ^18^F-FDG.

PET scans were analyzed visually and semi-quantitatively using standardised uptake value (SUV)_max_ measurements. SUV was expressed in terms of body weight *(*g/ml). Parameters such as the patient’s weight (kg) and height (cm), the radioactivity during injection (MBq), residual radioactivity (MBq) subsequent to the injection, starting time of the injection and the half-life of the radioisotope (taken as a standard 109.8 min for ^18^F-FDG) were calculated automatically by PET syngo VG50A software (Siemens Biograph Mctx; Siemens AG Healthcare Sector, Erlangen, Germany).

Two experienced nuclear medicine physicians blindly and independently reviewed the hybrid ^18^F-FDG-PET/CT scans as positive or negative for a primary tumour site. Every focal tracer uptake that deviated from physiological distribution was considered to be due to the disease spread. The background deviation and activity difference between the suspected lesion and the surrounding tissues was used to differentiate benign from malignant lesions; therefore, SUV_max_ >2.5 threshold was employed.

### Statistical analysis

Numeric results with a normal distribution, and for which parametric test methods were used, are expressed as the mean ± standard deviation, wheras those with a non-normal distribution, for which non-parametric test methods were used, are expressed as the median (min – max), and categorical results are presented as the number (%). All analyses were performed according to the intention-to-treat principle and all data analyses were performed using SPS 16.0 statistical software (SPSS, Inc., Chicago, IL, USA).

## Results

**Figure 1:**
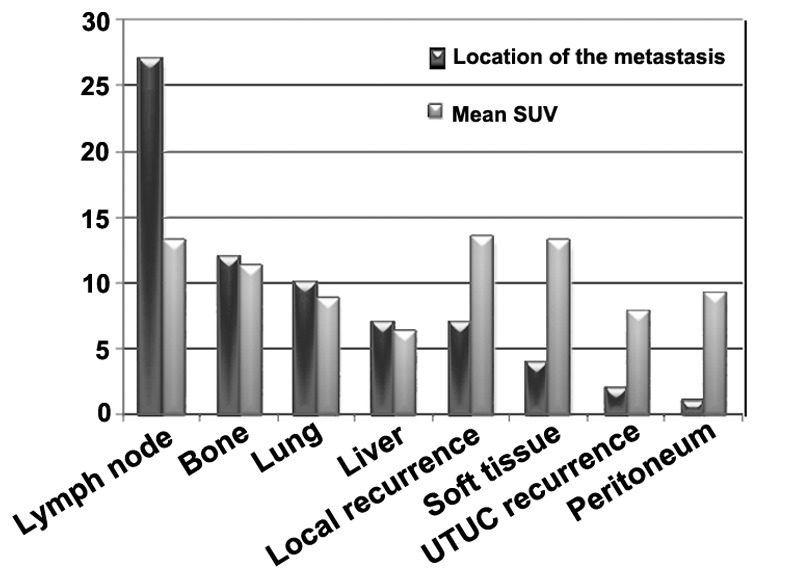
Distribution of the metastases and mean SUV of the metastatic foci. SUV, standardised uptake value; UTUC, upper urinary tract tumour.

^18^F-FDG-PET/CT scans showed negative findings in 13 patients (25.5%) and positive findings in 38 patients (74.5%). Seven patients (13.7%) had widespread metastases with high SUV (mean, 8.2; range 3.5–14.7) involving at least three organs (lungs or liver, bone, lymph nodes); 27 patients (52.9%) had lymph node metastasis (mean SUV, 12.5); 12 patients (23.5%) had bone metastasis (mean SUV, 11.2); 10 patients (19.6%) had lung metastasis (mean SUV, 8.3); seven patients (13.7%) had liver metastasis (mean SUV, 6.9); seven patients (13.7%) had local recurrence (mean SUV, 13.5); four patients (7.8%) had soft tissue metastasis (mean SUV, 13.2); two patients (3.9%) had recurrence of upper urinary tract tumour (UTUC) (mean SUV, 7.9); and one patient (1.9%) had peritonitis carcinomatosa (SUV, 9.2). The distribution of the metastases and mean SUV of the metastatic foci are summarised in Fig. 1.

Suspicious recurrent or metastatic lesions were confirmed by histopathology or by clinical follow-up. The results for sensitivity, specificity, positive predictive value (PPV), negative predictive value (NPV) and accuracy of ^18^F-FDG-PET/CT were 92, 83, 94, 77 and 90%, respectively.

## Discussion

MIBCs are associated with a poor prognosis. The survival rate decreases parallel to the stage of the disease and survival time decreases by more than half in the presence of metastatic disease. The five-year recurrence-free survival in node-positive patients who underwent cystectomy was 34–43%, which was considerably lower than that in patients without lymph-node involvement (5). In a surgery-only study, the five-year recurrence-free survival was 76, 74, 52 and 36% in patients with pT1, pT2, pT3 and pT4 tumours, respectively (5). Furthermore, according to a multi-institutional database of 888 consecutive patients undergoing radical cystectomy for bladder cancer, the five-year recurrence-free survival was 58% and the cancer-specific survival was 66% (10). At present, radical cystectomy involving the excision of pelvic lymph nodes is the gold standard treatment method in MIBC. Due to the fact that local recurrence and metastatic disease significantly decrease survival times in patients subsequent to radical cystectomy, it is of vital importance to establish an accurate diagnosis and provide prompt treatment at this stage of the disease. The restaging of the disease, minimizing false positives and false negatives and, more importantly, the recognition of metastatic disease, constitute the most important and realistic components in determining the treatment strategy. The detection of distant metastases and local recurrence continues to be a significant problem following radical cystectomy. Furthermore, there is considerable demand for a diagnostic test offering high sensitivity and specificity in predicting residual disease and monitoring treatment responses following radiotherapy and chemotherapy. ^18^F-FDG-PET/CT is the most important diagnostic tool that allows the processing of functional and anatomical images.

The most distinctive feature of the cancer tissue is that it shows a higher glucose metabolism than normal tissues (Warburg effect) (11). PET imaging with ^18^F-FDG, an analogue of glucose, tracks the glucose metabolism of tissues. The integral role of ^18^F-FDG-PET in oncology is indisputable. The hypermetabolism of malignancy is associated with an increased expression of cellular membrane glucose transporters and enhanced hexokinase enzymatic activity (12). A high uptake of ^18^F-FDG in cancerous lesions of transitional cell carcinomas was first demonstrated by Harney *et al* (13) in rats. Drieskens *et al* (14) found that metabolism-based anatomical information gathered by the addition of ^18^F-FDG-PET to CT provided high diagnostic accuracy in the pre-operative staging of invasive transitional cancers, particularly invasive bladder carcinoma. At present, ^18^F-FDG-PET combined with CT is an established standard for pre-operative staging and detecting metastatic lesions of bladder cancer (15–17).

^18^F-FDG in the systemic circulation undergoes glomerular filtration; however, it is excreted in the urine and not reabsorbed as glucose (18). This means that identifying kidney, ureter, bladder and prostate tumours is problematic (19). Another limitation is the poor ^18^F-FDG uptake by certain malignant neoplasms, such as renal, prostate and hepatocellular carcinomas. This has been attributed to their high glucose-6-phosphatase activity, the enzyme that converts ^18^F-FDG-6-phosphate back into ^18^F-FDG for excretion from the tumour cells (20). Another important reason for reduced uptake is that primary tumours may express low levels of glucose transporters, such as glucose transporter type 1, which are responsible for the accumulation of ^18^F-FDG (21).

Kosuda *et al* (22) used retrograde saline irrigation of the urinary bladder to remove ^18^F-FDG radioactivity; however, tracer activity was not able to be reduced to background levels and a 40% false-negative rate for the detection of recurrent or residual tumour in the bladder was reported. Diuresis has been shown to effectively decrease the background radioactivity in the urinary tract, thus facilitating the identification of hypermetabolic lesions on an ^18^F-FDG-PET scan (23). Anjos *et al* (24) reported a 54% sensitivity rate for ^18^F-FDG-PET/CT in the detection of malignant areas on the bladder wall of 11 patients with MIBC. A similar study by Harkirat *et al* (25) found a sensitivity of 86.7% and a specificity of 100% for ^18^FDG-PET/CT scans in the detection of primary lesions in 22 patients with MIBC. These two studies acquired late pelvic images with hyperhydration and diuresis. Parallel to these two studies late pelvic images were obtained with oral hyperhydration 2.5–3.0 h after the injection of ^18^F-FDG in 18 patients (35.2%) that underwent orthotopic ileal continent neobladder urinary diversion in the present study. This endeavour was to overcome the disadvantages posed by urinary excretion of ^18^F-FDG.

Lodde *et al* (26) performed ^18^F-FDG-PET/CT and forced diuresis in 44 patients with known MIBC and compared the findings with those from standard CT. It was demonstrated that ^18^F-FDG-PET/CT was more sensitive (85 vs. 77%) but less specific (25 vs. 50%) than CT alone for detecting primary tumours. The use of CT alone for the detection of MIBC exhibited a sensitivity of 46%, a specificity of 92% and an accuracy of 80%. Lodde *et al* (26) demonstrated that, regarding the detection of pelvic node metastasis, ^18^F-FDG-PET/CT was more sensitive than CT (57 vs. 33%) with a specificity and PPV of 100% for both imaging techniques. Drieskens *et al* (14) reported 60, 88 and 78% sensitivity, specificity and accuracy, respectively, for ^18^F-FDG-PET/CT in the detection of metastatic disease in 55 patients with MIBC. In a study by Swinnen *et al* (27) of 55 patients with MIBC in whom radical cystectomy was planned, the results of ^18^F-FDG-PET/CT scans were compared with the results of pathological examination following radical cystectomy and extended pelvic lymph node dissection. ^18^F-FDG-PET/CT achieved a sensitivity, specificity, and accuracy of 46, 97 and 84%, respectively. Kibel *et al* (16) studied 43 patients with a T2-3N0M0 stage tumour who underwent radical cystectomy, and reported a sensitivity of 70%, a specificity of 94%, a PPV of 78% and an NPV of 91% for ^18^F-FDG-PET/CT. Occult metastatic disease was found in seven out of 42 patients, and it was revealed that pre-operative ^18^F-FDG-PET/CT could affect the treatment selection prior to radical cystectomy (16). The same study also evaluated the association between PET findings and survival. The rate of 24-month recurrence-free survival was 24% in patients with positive PET findings and 55% in patients with negative PET findings. The disease-specific survival rates in these patients were 23 and 58%, respectively.

**Figure 2:**
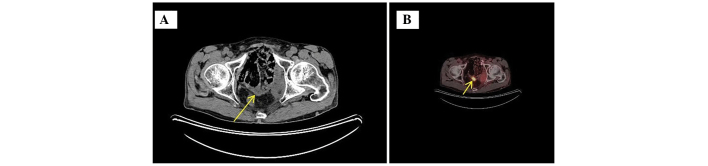
(A) Axial reconstruction showing CT imaging (the arrow indicates pelvic lymphadenopathy). (B) Axial ^18^F-fluorodeoxyglucose-positron emission tomography/CT scan showing the maximum standardised uptake value: 4.2 (the arrow indicates pelvic lymph node metastasis). CT, computed tomography.

**Figure 3:**
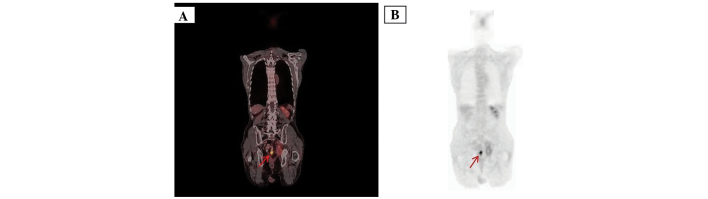
(A) Coronal ^18^F-fluorodeoxyglucose-positron emission tomography/computed tomography scan showing the maximum standardised uptake value: 4.2 (the arrow indicates pelvic lymph node metastasis). (B) Maximum intensity projection images of a patient (the arrow indicates pelvic lymph node metastasis).

**Figure 4:**
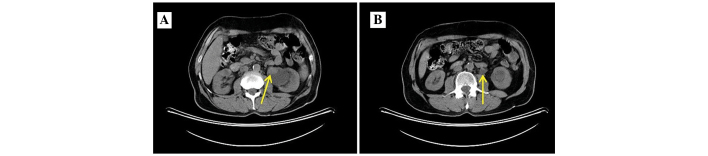
Computed tomography imaging. The axial reconstruction shows (arrow) the recurrence of upper urinary tract urothelial carcinoma in the (A) pelvis renalis and (B) proximal ureter.

**Figure 5:**
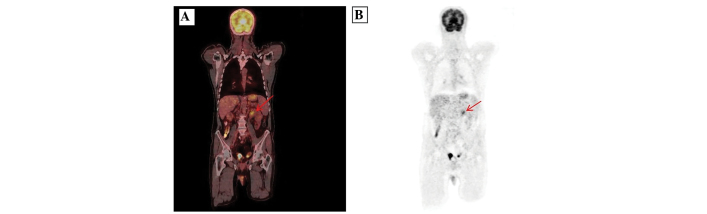
(A) Coronal ^18^F-fluorodeoxyglucose-positron emission tomography/computed tomography scan showing (arrow) the recurrence of upper urinary tract urothelial carcinoma (maximum standardised uptake, 7.9). (B) Maximum intensity projection images of a patient (the arrow indicates the recurrence of upper urinary tract urothelial carcinoma).

Parallel to these findings, we consider that urinary excretion of ^18^F-FDG partially eliminated the disadvantages of the method in patients that underwent radical cystectomy due to MIBC. In the present study, the use of ^18^F-FDG may have been disadvantageous in the 35% of patients (n=18) who underwent the ileal continent orthotopic neobladder procedure; however, the authors attempted to overcome these limitations by administering oral hyperhydration and acquiring late pelvic images. ^18^F-FDG activity in orthotopic continent neobladder may produce problems in detecting pelvic recurrence. The acquisition of late pelvic images and emptying the neobladder through catheterisation allowed visualisation of the pelvic lymph-node metastasis and recognition of the solitary lymph-node metastasis (Figs. 2 and 3). In the present study, the recurrences of UTUC could have produced problems due to urinary activity of ^18^F-FDG. UTUC recurrence was detected in only 3.9% (n=2) of the patients in the present study (Figs. 4 and 5). The high SUV (mean, 7.9) of these lesions allowed visualisation on the detector. The detection of low-grade UTUC with low metabolism continues to be a significant problem using ^18^F-FDG-PET/CT scans. Although survival data are not available in the present study, other parameters are in line with those reported in the literature (14–16). According to these data from the literature, ^18^F-FDG-PET/CT scans prior and subsequent to cystectomy are considered to play an important role in the planning of treatment strategies.

Apolo *et al* (15) evaluated the role of ^18^F-FDG-PET/CT in a series of 47 patients with metastatic MIBC and reported a sensitivity of 88% and a specificity of 87%. The patient-based analyses found that ^18^F-FDG-PET/CT scans may change the treatment plan in 68% of the patients due to a 40% higher detection rate compared with that of conventional CT and MRI. ^18^F-FDG-PET/CT has additionally been found to provide diagnostic data relevant to the clinical management of the disease due to its higher sensitivity and specificity in metastatic MIBC (28). Jadvar *et al* (29) retrospectively evaluated the diagnostic performance of PET/CT in patients with MIBC, and they reported that the method changed the clinical management in 17% of the patients. In a meta-analysis by Lu *et al* (17), ^18^F-FDG-PET/CT scans were found to provide sufficient diagnostic accuracy in the staging and restaging of patients with MIBC and metastatic bladder cancer; however, ^18^F-FDG-PET/CT scans did not show sufficient diagnostic performance in the detection of primary bladder cancer on the bladder wall. The study revealed that the method may not provide sufficient data regarding the T stage of the bladder and detrusor lesions due to urinary excretion of ^18^F-FDG, but ^18^F-FDG-PET/CT can be used for staging purposes and the detection of metastatic disease (17). ^18^F-FDG-PET/CT scans provide valuable data in the detection of metastatic disease and in the evaluation of the response to systemic chemotherapy and detection of residual disease. In addition, ^18^F-FDG-PET/CT is used to evaluate insufficient response to cisplatin-based chemotherapy in patients with lung and lymph-node metastases after radical cystectomy.

Recently, Goodfellow *et al* (30) evaluated the efficiency of ^18^F-FDG-PET/CT compared with CT in patients in whom radical cystectomy was planned due to MIBC. Although PET/CT had certain advantages in detecting distant metastases, CT offered 45% sensitivity and 98% specificity in detecting pelvic lymph nodes; the combination of ^18^F-FDG-PET/CT increased sensitivity to 69%, while the specificity was 95%. It was suggested that the use of ^18^F-FDG-PET/CT instead of CT provided a slight improvement in the pre-operative diagnosis of MIBC, but that improvement did not justify the increasing costs of the diagnostic workup; therefore, it was recommended that this method be used only in selected patients (30). In a recent study, Mertens *et al* (31) evaluated the association between ^18^F-FDG-PET/CT results and mortality in patients with MIBC. In the study (n=211), the median follow-up period was 18 months and the disease-specific survival was 50 months in PET-negative patients; this rate decreased to 16 months in PET-positive patients. The presence of extravesical disease was found to be an independent prognostic factor for mortality in PET-positive patients (31).

Due to the physiological activity of ^18^F-FDG in the urinary tract, ^11^C-choline, ^11^C-acetate and ^11^C-methionine have been used in an attempt to overcome the diagnostic limitations of ^18^F-FDG-PET/CT. ^11^C-choline is minimally excreted in the urine and is incorporated into tumour cells by conversion into ^11^C-phosphorlycholine, which is trapped inside the cell (32). A study that evaluated ^11^C-choline PET/CT in patients with MIBC reported a sensitivity, specificity, PPV, NPV and accuracy of 58, 66, 39, 81 and 64%, respectively (33). A study by Golan *et al* compared ^11^C-choline-PET/CT with ^18^F-FDG-PET/CT reported that ^11^C-choline was not advantageous compared with other methods (34). In the study by Golan *et al* (34), which evaluated 51 lesions with abnormal activity in 20 patients, the PPV for all lesions was found to be 84.7% for ^11^C-choline-PET/CT and 90.7% for ^18^F-FDG-PET/CT. In the evaluation of extravesical lesions, the PPV was 79.4% and 88.2%, respectively. Despite the disadvantage of partial histopathological correlation, ^11^C-choline-PET/CT was not found to be superior to ^18^F-FDG-PET/CT in detecting metastatic bladder carcinoma (34). The diagnostic performances of ^18^F-FDG-PET/CT and ^11^C-choline-PET/CT reported in the literature are summarised in Table II. ^11^C-methionine uptake is proportional to the amino acid transport and, to a certain extent, protein synthesis. In cancer, methionine levels have been correlated with the amount of viable tumour tissue (33). Ahlström *et al* (35) found that ^11^C-methionine was superior to ^18^F-FDG in patients with MIBC. Schöder *et al* (36) investigated the utility of ^11^C-acetate-PET/CT for the staging of MIBC and the assessment of response subsequent to neoadjuvant chemotherapy. A total of 17 patients underwent ^11^C-acetate-PET/CT prior to radical cystectomy and pelvic lymph-node dissection. It was concluded that ^11^C-acetate-PET/CT offered high sensitivity in the detection of lymph-node metastases; however, inflammation and granulomatous infections and false-positive results following intravesical Bacillus Calmette-Guerin therapy were reported as the limitations of this method (36).

The metabolic rate of low-grade transitional cell carcinomas is close to that of normal tissues. The increased glucose metabolism in patients with high-grade MIBC allows visualisation of the lesions on the detector due to increased ^18^F–FDG uptake.

False-positive or false-negative results in ^18^F-FDG uptake cannot be explained solely by the glucose metabolism of tumour tissue. Studies have demonstrated that ^18^F-FDG-PET/CT scans can provide information only in the presence of a high number of tumour cells with abnormal glucose metabolism (10^4^*–*10^7^) (11,15,16). Such diagnostic failures are particularly important in solid organ metastasis, such as in the lungs and liver. In general, ^18^F-FDG-PET/CT cannot accurately evaluate metastasis measuring <5 mm in size. It is unknown why lung lesions below this threshold do not produce high SUVs. This could be caused by motion artifacts and low metabolic activity of the metastatic lesion. Reducing the motion artifacts using certain techniques, achieving an enhanced spatial resolution and finding higher cut-off SUV values for such lesions could increase diagnostic accuracy (37).

The findings of PET/CT scans must be verified by histopathological work-up in order to confirm disease recurrence. Theoretically, this remains the gold standard. In daily practice, however, this is seldom possible due to clinical reasons, the feasibility of the procedure and the effective advantages of this approach in the absence of a radical surgical intent. In the present study histological confirmation was available for 15 patients, while the remainder relied on the comparison with clinical and radiological findings.

The limitation of the present study was its retrospective nature. Selection bias may have been present as it is likely that only those patients with MIBC and suspected to have recurrence were referred for PET/CT.

In conclusion, ^18^F-FDG-PET/CT images provide complementary structural-metabolic information and have the potential to significantly reduce the false positives of PET and CT performed separately. Despite the limitations of the present study, due to the retrospective type of analysis and the absence of systematic histological confirmation of pathological uptake, the results were in agreement with those of previous studies and suggest that ^18^F-FDG-PET/CT is characterised by a high sensitivity and PPV and could be useful in restaging patients with MIBC following radical cystectomy. This procedure could play an important role in rendering decisions regarding radiotherapy, chemotherapy and post-operative follow-up.

## Figures and Tables

**Table I:** Baseline characteristics of the patients (n=51).

Patient parameter	Value
Age, years	
Mean	62
Range	40–82
Gender, n (%)	
Male	45 (88.2)
Female	6 (11.8)
Histological type, n (%)	
Urothelial carcinoma	47 (92.2)
Squamous cell carcinoma	1 (1.9)
Unknown	3 (5.9)
Urinary diversion type, n (%)	
Bricker ileal conduit	30 (58.8)
Hautmann orthotopic neobladder	18 (35.2)
Ureterocutaneostomy	3 (6.0)

**Table II:** The diagnostic performance of ^18^F-FDG-PET/CT and ^11^C-choline-PET/CT studies in the literature.

First author (reference)	Modality	n	Status of the BCa	Sensitivity (%)	Specificity (%)	PPV (%)	NPV (%)	Accuracy (%)
Harkirat (25)	^18^F-FDG-PET/CT	22	Primary BCa	86.7	100	-	-	-
Lodde (26)	^18^F-FDG-PET/CT	44	Primary BCa	77	50	100	-	-
Drieskens (14)	^18^F-FDG-PET/CT	55	Metastatic BCa	60	88	-	-	78
Apollo (15)	^18^F-FDG-PET/CT	47	Metastatic BCa	88	87	-	-	-
Swinnen (27)	^18^F-FDG-PET/CT	55	MIBC, before cystectomy	46	97	-	-	84
Goodfellow (30)	^18^F-FDG-PET/CT	-	MIBC, before cystectomy	68	95	-	-	-
Maurer (33)	^11^C-choline-PET/CT	44	MIBC, before cystectomy	58	66	39	81	64
Kibel (16)	^18^F-FDG-PET/CT	43	MIBC, before cystectomy	70	94	78	91	-
Present study	^18^F-FDG-PET/CT	51	MIBC, after cystectomy	92	83	94	77	90

^18^F-FDG-PET/CT, ^18^F-fluorodeoxyglucose-positron emission tomography/computed tomography; PPV, positive predictive value; NPV, negative predictive value; BCa, bladder cancer; MIBC, muscle-invasive bladder carcinoma.
